# Availability of resources to treat sepsis in Brazil: a random sample of Brazilian institutions

**DOI:** 10.5935/0103-507X.20190033

**Published:** 2019

**Authors:** Leandro Utino Taniguchi, Luciano Cesar Pontes de Azevedo, Fernando Augusto Bozza, Alexandre Biasi Cavalcanti, Elaine Maria Ferreira, Fernanda Sousa Angotti Carrara, Juliana Lubarino Sousa, Reinaldo Salomão, Flávia Ribeiro Machado

**Affiliations:** 1 Disciplina de Emergências Clínicas, Hospital das Clínicas, Faculdade de Medicina, Universidade de São Paulo - São Paulo (SP), Brasil.; 2 Hospital Sírio-Libanês - São Paulo (SP), Brasil.; 3 Brazilian Research in Intensive Care Network - São Paulo (SP), Brasil.; 4 Instituto Latino Americano da Sepse - São Paulo (SP), Brasil.; 5 Instituto Nacional de Infectologia Evandro Chagas, Fundação Oswaldo Cruz - Rio de Janeiro (RJ), Brasil.; 6 Instituto D'Or de Pesquisa e Ensino - Rio de Janeiro (RJ), Brasil.; 7 Instituto de Pesquisa, HCor-Hospital do Coração - São Paulo (SP), Brasil.; 8 Departamento de Moléstias Infecciosas, Universidade Federal de São Paulo - São Paulo (SP), Brasil.; 9 Departamento de Anestesiologia, Dor e Terapia Intensiva, Universidade Federal de São Paulo - São Paulo (SP), Brasil.

**Keywords:** Critical care, Health resources, Sepsis/epidemiology, Therapeutics, Epidemiological monitoring, Brazil/epidemiology, Developing countries, Intensive care units, Cuidados críticos, Recursos em saúde, Sepse/epidemiologia, Terapêutica, Monitoramento epidemiológico, Brasil/epidemiologia, Países em desenvolvimento, Unidades de terapia intensiva

## Abstract

**Objective:**

To characterize resource availability from a nationally representative random sample of intensive care units in Brazil.

**Methods:**

A structured online survey of participating units in the Sepsis PREvalence Assessment Database (SPREAD) study, a nationwide 1-day point prevalence survey to assess the burden of sepsis in Brazil, was sent to the medical director of each unit.

**Results:**

A representative sample of 277 of the 317 invited units responded to the resources survey. Most of the hospitals had fewer than 500 beds (94.6%) with a median of 14 beds in the intensive care unit. Providing care for public-insured patients was the main source of income in two-thirds of the surveyed units. Own microbiology laboratory was not available for 26.8% of the surveyed intensive care units, and 10.5% did not always have access to blood cultures. Broad spectrum antibiotics were not always available in 10.5% of surveyed units, and 21.3% could not always measure lactate within three hours. Those institutions with a high resource availability (158 units, 57%) were usually larger and preferentially served patients from the private health system compared to institutions without high resource availability. Otherwise, those without high resource availability did not always have broad-spectrum antibiotics (24.4%), vasopressors (4.2%) or crystalloids (7.6%).

**Conclusion:**

Our study indicates that a relevant number of units cannot perform basic monitoring and therapeutic interventions in septic patients. Our results highlight major opportunities for improvement to adhere to simple but effective interventions in Brazil.

## Abstract (pt)

**Objetivo:**

Caracterizar a disponibilidade de recursos a partir de amostra aleatória representativa das unidades de terapia intensiva do Brasil.

**Métodos:**

Realizou-se um questionário estruturado *on-line* para ser respondido pelo diretor médico de cada unidade participante do estudo SPREAD (*Sepsis PREvalence Assessment Database*), um estudo de prevalência de um único dia para avaliar o ônus da sepse no Brasil.

**Resultados:**

Uma amostra representativa de 277 das 317 unidades convidadas participou por meio de resposta ao questionário estruturado. Em sua maior parte, os hospitais participantes tinham menos que 500 leitos (94,6%), com mediana de 14 leitos na unidade de terapia intensiva. A principal fonte de recursos financeiros para dois terços das unidades pesquisadas era o atendimento de pacientes do sistema público de saúde. Não havia disponibilidade de laboratório de microbiologia próprio em 26,8% das unidades de terapia intensiva pesquisadas, e 10,5% geralmente não tinham acesso à realização de hemoculturas. Em 10,5% das unidades pesquisadas geralmente não estavam disponíveis antibióticos de amplo espectro, e 21,3% das unidades geralmente não podiam obter mensurações de lactato dentro de 3 horas. As instituições com alta disponibilidade de recursos (158 unidades; 57%) eram, em geral, maiores e atendiam principalmente pacientes do sistema de saúde privado. As unidades sem alta disponibilidade de recursos geralmente não dispunham de antibióticos de amplo espectro (24,4%), vasopressores (4,2%) e cristaloides (7,6%).

**Conclusão:**

Um número importante de unidades não tem condições para realizar intervenções básicas de monitoramento e terapêutica em pacientes sépticos. Nossos resultados salientam importantes oportunidades que o Brasil tem para melhorar, em termos de adesão a intervenções simples, porém eficazes.

## INTRODUCTION

Sepsis is a global health priority, as recently stated by the World Health Organization.^(1,2)^ Current extrapolation based on a recent systematic review estimates 31.5 million cases of sepsis per year worldwide, with a potential of 5.3 million deaths. However, this extrapolation was based on data from high-income countries.^(3)^ Since more than 80% of the world's population lives in low- and middle-income countries (LMICs), where resource limitations are frequent, the lethality rates are likely much higher.^(1,4,5)^ The lack of reliable data on resource availability from LMICs is also noteworthy.^(6)^ Although some information is available,^(7-10)^ these studies are largely single-center descriptions or questionnaire-based surveys without random sampling, which might induce selection bias.

Brazil is a middle-income country according to the World Bank^(11)^ with an estimated population of approximately 209 million people;^(12)^ some data suggest an increase in sepsis-related deaths from 2002 to 2010 in Brazil.^(13)^ The Sepsis PREvalence Assessment Database study (SPREAD), a nationwide 1-day point prevalence survey of Brazilian intensive care units (ICU), observed an ICU sepsis incidence of 36.3 cases per 1000 patient-days and an alarming hospital mortality of 55.7%. Low resource availability was independently associated with mortality (odds ratio 1.67, p = 0.045).^(14)^ Since this survey generated a nationally representative random sample from Brazilian ICUs with a description of institution infrastructure, resource availability, and ICU organizational aspects from participating units, this information is more representative than previous small convenience cohorts.^(15,16)^

Thus, we performed a post hoc analysis of the SPREAD database to characterize and compare the resource availability of participating units. Patient characterization and outcomes were described in the original publication.^(14)^

## METHODS

The SPREAD study was conducted as a 1-day, prospective, point prevalence study to assess the epidemiology of sepsis in adult ICUs in Brazil.^(14)^ A stratified random sample of all Brazilian adult ICUs was generated from the *Associação de Medicina Intensiva Brasileira* (AMIB) 2010 Census.^(17)^ It comprised 2,623 ICUs with 28,849 beds. After excluding neonatal and pediatric ICUs, cardiac care units, and burn units, a list of 1,690 ICUs and 19,316 eligible beds remained.

Our sampling method is explained in the original publication.^(14)^ Briefly, we created similarly sized strata, each composed of 100 - 500 ICU beds to enhance the representativeness of our random selection of ICUs. Based on the AMIB list, we produced a sampling frame initially stratified by geographic region and size of the cities (considering the location, whether in capital cities or the countryside). Each stratum was then stratified by the hospitals' main source of income (serving public or privately insured individuals) and ICU size (ten or fewer beds *versus* more than ten beds), finally generating 40 strata. We applied the "randomize" (RAND) function in Excel 2010, which generates random numbers for ICUs within each stratum and sequentially contacted their medical directors by telephone and email, inviting them to participate in the study. This study was approved by the research ethics committee at the coordinating center (*Universidade Federal de São Paulo*, Brazil) under the number CAAE: 04719512.0.1001.5505. Informed consent was waived because of the observational nature of the study.

### Participants and survey instrument

We assessed the ICU organizational factors and institution resource availability through a self-reported, questionnaire-based web survey (Supplementary material). The medical director of each ICU answered the questionnaire before study entry and patient data collection. No financial incentive to complete the survey or to participate in the SPREAD study was offered.

The questionnaire was designed by the Steering Committee of the SPREAD study and contained 97 items, which were grouped into eight main categories (general information, hospital facilities, use of clinical protocols and availability of drugs, monitoring tools, laboratory exams, equipment and disposables). The "general information" section had two open-ended responses ('number of hospital beds' and 'number of ICU beds' in the institution), which were later categorized by the study investigators. The responses were classified as 'yes', 'no' and 'I don't know' for the "hospital facilities" section; 'yes, a managed protocol', 'yes, but not managed', 'no' and 'I don't know' for the "clinical protocols" section; and 'always', 'most of the time', 'in the minority of times', 'never', and 'I don't know' for the other sections. No missing variables were allowed. To assess the most relevant resources, the Steering Committee selected eight items using an informal Delphi process before performing any analyses, under the premise that they would be required to comply with the Surviving Sepsis Campaign 6-h bundle.^(18)^ These eight items were: blood gas analysis within 3 hours; lactate results within 3 hours; blood, urine and tracheal aspirate (quantitative or qualitative) cultures; antibiotics both for gram-negative (a third-generation cephalosporin plus carbapenems or piperacillin/tazobactam) and gram-positive coverage (vancomycin, teicoplanin or linezolid); crystalloids; noradrenaline; central venous catheter (single or double lumen); and availability for central venous pressure measurement.

### Study variables and data analysis

Since previous literature^(19)^ and data from the SPREAD^(14)^ study suggested that compliance with the 6-h bundle was associated with lower hospital mortality, we categorized the units according to the availability of all eight items ('high resource availability' when all 8 items were always available and 'without high resource availability' when one or more of the 8 items were not always available). For the analysis, we considered the units as having the resource available only when the answer was 'always'.^(7)^ We also compared the microbiology analysis resource availability and the possibility to administer broad-spectrum antibiotics (defined as antibiotics for both gram-negative and gram-positive coverage as defined in the 8-item section). The possibility to adhere to the Surviving Sepsis Campaign recommendations labeled as 'strong' and the recent 1-h bundle were evaluated.^(20)^

Continuous data are presented as the median (25^th^ - 75^th^ percentile) and were compared using the Mann-Whitney U test. Categorical variables are presented as counts and rates or percentages and were compared with the chi-squared test. P-values < 0.05 were considered statistically significant. The software Statistical Package for Social Science (SPSS), version 20 (SPSS Inc., Chicago, IL, USA) was used for the statistical analysis.

## RESULTS

Of the 368 contacted ICUs, 317 were eligible and 13 (4%) refused to participate. Of the 317 eligible units, 277 (87%) answered the resources survey and are further described in the present publication. Most of the hospitals were small- to medium-sized (262 hospitals, 94.6%) with a median of 14 (9 - 30) ICU beds. In two-thirds of hospitals, the main source of income was the care for public-insured patients (169 ICUs, 61%). The geographic distribution of participating institutions paralleled the Brazilian population distribution among regions. The nurse/patient ratio was 0.13 (0.10 - 0.19), and the nurse technician/patient ratio was 0.5 (0.5 - 0.5). Although most hospitals had emergency departments (247 hospitals, 89.5%) and operating rooms (274 hospitals, 98.9%), only 73.2% had their own microbiology laboratory, and almost half lacked their own blood bank (Table 1). Twenty-nine units (10.5%) did not always have the possibility to administer broad-spectrum antibiotics, nine (3.2%) did not always have access to crystalloids and five (1.8%) did not always have vasopressors available (neither norepinephrine nor dopamine) (Table 2). In twenty-nine institutions (10.5%), access to blood cultures was not always possible, and 59 (21.3%) could not always measure lactate levels within three hours (Table 3).

**Table 1:** General institution characteristics

Variable	Global (n = 277)	High resource availability (n = 158)	Without high resource availability (n = 119)	p value*
Hospital size				0.669
≤ 100 beds	77 (27.8)	41 (25.9)	36 (30.3)	
101 to 500 beds	185 (66.8)	109 (69.0)	76 (63.9)	
> 500 beds	15 (5.4)	8 (5.1)	7 (5.9)	
ICU beds				< 0.001
≤ 10	115 (41.5)	48 (30.4)	67 (56.3)	
11 to 50	130 (46.9)	89 (56.3)	41 (34.5)	
> 50	32 (11.6)	21 (13.3)	11 (9.2)	
ICU beds (number)	14 (9 - 30)	19 (10 - 35.25)	10 (8 - 20)	< 0.001
Hospital location				< 0.001
Capitals	140 (50.5)	100 (63.3)	40 (33.6)	
Countryside	137 (49.5)	58 (36.7)	79 (66.4)	
Hospital characteristics				< 0.001
Private health system	108 (39.0)	83 (52.5)	25 (21.0)	
SUS	169 (61.0)	75 (47.5)	94 (79.0)	
Geographic region				0.095
Southeast	138 (49.8)	86 (54.4)	52 (43.7)	
South	46 (16.6)	24 (15.2)	22 (18.5)	
Middle-West	19 (6.9)	14 (8.9)	5 (4.2)	
Northeast	53 (19.1)	24 (15.2)	29 (24.4)	
North	21 (7.6)	10 (6.3)	11 (9.2)	
Teaching status				0.051
University	57 (20.6)	26 (16.5)	31 (26.1)	
Non-university	220 (79.4)	132 (83.5)	88 (73.9)	
Healthcare staff				
Nurse/patient ratio	0.13 (0.10 - 0.19)	0.13 (0.10 - 0.20)	0.14 (0.10 - 0.18)	0.510
Nurse technician/patient ratio	0.5 (0.5 - 0.5)	0.5 (0.5 - 0.5)	0.5 (0.5 - 0.5)	0.004
Physician/patient ratio (day)	0.13 (0.10 - 0.17)	0.13 (0.10 - 0.17)	0.13 (0.10 - 0.16)	0.852
Physician/patient ratio (night)	0.11 (0.10 - 0.14)	0.10 (0.10 - 0.13)	0.11 (0.10 - 0.14)	0.043
Hospital facilities				
Emergency	247 (89.5)	140 (88.6)	107 (89.9)	0.842
Operating theater	274 (98.9)	157 (99.4)	117 (98.3)	0.404
Own blood bank	162 (59.1)	89 (56.3)	73 (61.3)	0.342
Own laboratory	232 (83.8)	136 (86.1)	96 (80.7)	0.227
Own microbiology	202 (73.2)	125 (79.1)	77 (64.7)	0.010

ICU - intensive care unit; SUS - Brazilian public health system.

* Chi-square or Mann-Whitney U tests between institutions with high resource availability compared to those without. The results are expressed as numbers (%) or the median (25%-75% percentiles).

**Table 2:** Availability of medicines according to the institution availability of resources

Variable	Global (n = 277)	High resource availability (n = 158)	Without high resource availability (n = 119)	p value*
Antibiotics (answer: always)				
3^rd^ generation cephalosporins	263 (94.9)	158 (100.0)	105 (88.2)	< 0.001
4^th^ generation cephalosporins	249 (89.9)	157 (99.4)	92 (77.3)	< 0.001
Piperacillin/tazobactam	230 (83.0)	155 (98.1)	75 (63.0)	< 0.001
Carbapenems	246 (88.8)	157 (99.4)	89 (74.8)	< 0.001
Vancomycin	257 (92.8)	158 (100)	99 (83.2)	< 0.001
Linezolid	141 (51.5)	105 (66.5)	36 (30.3)	< 0.001
Macrolide	217 (78.9)	140 (88.6)	77 (64.7)	< 0.001
Echinocandins	113 (41.4)	96 (60.8)	17 (14.3)	< 0.001
Tigecycline	112 (41.3)	91 (57.6)	21 (17.6)	< 0.001
Other drugs (answer: always)				
Hydrocortisone	267 (96.4)	157 (99.4)	110 (92.4)	0.002
Crystalloids	268 (96.8)	158 (100.0)	110 (92.4)	< 0.001
Albumin	212 (76.5)	138 (87.3)	73 (61.3)	< 0.001
Norepinephrine	272 (98.2)	158 (100.0)	114 (95.8)	0.009
Dopamine	260 (93.9)	152 (96.2)	108 (90.8)	0.062
Dobutamine	271 (97.8)	158 (100.0)	113 (95.0)	0.004
Adrenaline	272 (98.2)	158 (100.0)	114 (95.8)	0.009
Vasopressin	138 (50.5)	103 (65.2)	35 (29.4)	< 0.001
Red blood cell within 6 hours	249 (89.9)	149 (94.3)	100 (84.0)	0.005

* Chi-square test between institutions with high resource availability compared to those without. The results are expressed as numbers (%).

**Table 3:** Availability of laboratory exams according to the institution availability of resources

Variable	Global (n = 277)	High resource availability (n = 158)	Without high resource availability (n = 119)	p value*
Laboratory (answer: always)				
Direct microscopy/Gram	0231 (83.4)	150 (94.9)	81 (68.1)	< 0.001
Blood culture	248 (89.5)	158 (100.0)	90 (75.6)	< 0.001
Respiratory secretions (qualitative)	210 (76.6)	151 (95.6)	59 (49.6)	< 0.001
Respiratory secretions (quantitative)	196 (71.8)	143 (90.5)	53 (44.5)	< 0.001
Urine culture	250 (90.3)	158 (100.0)	92 (77.3)	< 0.001
Blood gas analysis within 3 hours	254 (91.7)	158 (100.0)	96 (80.7)	< 0.001
Lactate within 3 hours	218 (78.7)	158 (100.0)	50 (60.4)	< 0.001
C-reactive protein	246 (89.1)	151 (95.6)	95 (79.8)	< 0.001
Procalcitonin	38 (14.5)	28 (17.7)	10 (8.4)	0.026

* Chi-square test between institutions with high resource availability compared to those without. The results are expressed as numbers (%).

The units with high resource availability were usually larger, located in capitals and cared for patients from the private health system compared to those without high resource availability. They also had a higher number of nurse technicians per patient but a similar number of registered nurses and daily physicians per patient (Table 1). Among the units without high resource availability, 24.4% did not have broad-spectrum antibiotics, 4.2% did not have vasopressors and 7.6% did not have crystalloids (Table 2). Microbiology laboratory resources, lactate measures, disposables, equipment and monitoring devices availability were systematically different between these two types of units (Tables 3 and 4). Protocolized care was also different (Table 5). Institutions with lower access to microbiology analyses also had lower access to broad-spectrum antibiotics (Table 6).

**Table 4:** Availability of disposables and monitoring/diagnosis devices according to the institution availability of resources

Variable	Global (n = 277)	High resource availability (n = 158)	Without high resource availability (n = 119)	p value*
Disposables (answer: always)				
Oxygen mask/nasal probes	271 (97.8)	155 (98.1)	116 (97.5)	0.75
Noninvasive ventilation	241 (87.3)	148 (93.7)	93 (78.2)	< 0.001
Mechanical ventilator	264 (95.6)	154 (97.5)	110 (92.4)	0.05
Tracheal tube	277 (100.0)	158 (100.0)	119 (100.0)	1.00
Infusion pump	273 (98.5)	157 (99.4)	116 (97.5)	0.152
Bedside RRT	239 (86.5)	151 (95.6)	88 (73.9)	< 0.001
Urinary catheter	274 (98.9)	158 (100.0)	116 (97.5)	0.045
Enteral tube feeding	268 (96.7)	157 (99.4)	111 (93.3)	0.005
Peripheral catheters	273 (98.5)	158 (100.0)	115 (96.6)	0.020
Central line catheters	267 (97.4)	151 (95.6)	95 (79.8)	< 0.001
Monitoring devices (answer: always)				
Automatic blood pressure	267 (96.4)	157 (99.4)	110 (92.4)	0.002
Invasive blood pressure	161 (58.1)	129 (81.6)	32 (26.9)	< 0.001
CVP	214 (77.3)	158 (100.0)	56 (47.1)	< 0.001
Noninvasive cardiac output	61 (22.1)	49 (31.0)	12 (10.1)	< 0.001
Pulmonary artery catheter	79 (28.6)	67 (42.4)	12 (10.1)	< 0.001
Continuous SvO_2_	26 (9.6)	24 (15.2)	2 (1.7)	< 0.001
Bedside X-ray	262 (94.5)	156 (98.7)	106 (89.1)	< 0.001
Bedside ultrasound	142 (51.2)	103 (65.2)	39 (32.8)	< 0.001
Bedside echocardiography	131 (47.2)	98 (62.0)	33 (27.7)	< 0.001
Computed tomography	223 (80.5)	142 (89.9)	81 (68.1)	< 0.001

RRT - renal replacement therapy; CVP - central venous pressure; SvO_2_ - central venous oxygen saturation.

* Chi-square test between institutions with high resource resources compared to those without. The results are expressed as numbers (%).

**Table 5:** Clinical management according to the institution availability of resources

Variable	Global (n = 277)	High resource availability (n = 158)	Without high resource availability (n = 119)	p value*
Management (answer: always + almost always)				
Invasive blood pressure in shock	199 (71.8)	134 (84.8)	65 (54.6)	< 0.001
CVP in shock	237 (85.6)	143 (90.5)	94 (79.0)	0.007
CVP in hyperlactatemia	217 (78.3)	141 (89.2)	106 (63.9)	< 0.001
Fluid responsiveness	83 (30.3)	65 (41.1)	18 (15.3)	< 0.001
SvcO_2_ in shock	231 (83.4)	138 (87.3)	93 (78.2)	0.042
SvcO_2_ in hyperlactatemia	218 (78.7)	137 (86.7)	81 (68.6)	< 0.001
Lactate in severe sepsis suspicious	247 (89.2)	155 (98.1)	92 (77.3)	< 0.001
Protocolized care				
Sepsis	228 (82.3)	140 (88.6)	88 (73.9)	0.002
Glycemic control	255 (92.1)	149 (94.3)	106 (89.1)	0.111
Sedation	227 (81.9)	133 (84.2)	94 (79.0)	0.267
MV weaning	239 (86.3)	139 (88.0)	100 (84.0)	0.345
Nutrition	214 (77.8)	134 (84.8)	80 (68.4)	0.001

CVP - central venous pressure; SvO_2_ - central venous oxygen saturation; MV - mechanical ventilation.

* Chi-square test between institutions with high resource availability compared to those without. The results are expressed as numbers (%).

**Table 6:** Microbiology resources according to antibiotic availability

Variable	Has broad-spectrum ATB availability (n = 248)	Does not have broad-spectrum ATB availability (n = 29)	p value*
Laboratory (answer: always)			
Direct microscopy/Gram	215 (86.7)	16 (55.2)	< 0.001
Blood culture	231 (93.1)	17 (58.6)	< 0.001
Respiratory secretions (qualitative)	196 (79.0)	14 (48.3)	0.001
Respiratory secretions (quantitative)	185 (74.6)	11 (37.9)	< 0.001
Urine culture	233 (94.0)	17 (58.6)	< 0.001

ATB - antibiotic. Adequate broad-spectrum ATB availability - antibiotics both for gram-negative (a third-generation cephalosporin plus carbapenens or piperacillin/tazobactam) and gram-positive coverage (vancomycin, teicoplanin or linezolid).

* Chi-square test between institutions with high resource availability compared to those without. The results are expressed as numbers (%).

Among all units, 214 (77.3%) were able to adhere to the 1-h bundle, and 219 (79.1%) were able to adhere to the 'strong' recommendations from the Surviving Sepsis Campaign. Notable differences were observed between the units with high resource availability and those without (Table 7).

**Table 7:** Possibility to adhere to the 1-hour bundle and to the Surviving Sepsis Campaign ‘strong’ recommendations

Variable	Global (n = 277)	High resource availability (n = 158)	Without high resource availability (n = 119)	p value*
1-hour bundle	214 (77.3)	158 (100.0)	56 (47.1)	< 0.001
‘Strong’ recommendations	219 (79.1)	139 (88.0)	80 (67.2)	< 0.001

* Chi-square test between institutions with high resource availability compared to those without. The results are expressed as numbers (%).

## DISCUSSION

The results of our nationwide, random, self-reported, questionnaire-based survey of a representative sample of Brazilian adult ICUs indicate that a substantial number of units cannot perform some basic monitoring (e.g., lactate measurement) and therapeutic interventions (e.g., broad-spectrum antibiotics) in septic patients. Human resources, medicine, equipment and laboratory availability are systematically different when comparing units with high resource availability (as a surrogate to adhere to the 6-h bundle) and those without. Almost one-quarter of ICUs could not comply with the 1-h bundle because of the lack of resources rather than the short time frame. Our results are relevant both for our country and as a framework to study the availability of resources in LMICs.

Information on resource availability in LMICs is scarce and is mostly limited to single-center data instead of representative national samples.^(21)^ In the ICON study, only 8.5% of participating centers were from low-income countries. Notably, a higher in-hospital risk of death was independently associated with a lower national income.^(22)^ One of the possible explanations is the difference in equipment, training and resource availability among centers. These differences might affect the possibility to adhere to first-line treatments. In fact, in the SPREAD study, lower resource availability was associated with a higher mortality in the multivariate analysis.^(14)^ Conversely, the IMPRESS study suggests that compliance with evidence based-bundles is associated with a lower mortality.^(19)^ Since resource availability in critical care seems to be associated with outcomes, the health care inequalities of LMICs, albeit notorious,^(23)^ should be further characterized.

Previous publications have suggested that the implementation of sepsis bundles in some LMICs is compromised because the availability of equipment, drugs and disposables are inadequate.^(7-10)^ Baelani et al. reported that in some African countries, 16.3% of units could implement the resuscitation bundles, which is much lower than the percentage in high-income countries (93.2%).^(7)^ Although our results for the 1-h bundle were better than those from African units, only 77.3% of our institutions had availability of required resources. When evaluating the individual components of the 1-h bundle in our study, it is particularly striking that some key therapeutic interventions are not always available (e.g., 3.2% lacked crystalloids, 1.8% lacked vasopressors, and 10.5% did not have broad-spectrum antibiotics). The unavailability of antibiotics is particularly worrisome since 60% of observed infections in SPREAD patients were health-care associated infections (which usually occur due to multiresistant microorganisms). We also observed a relationship between microbiology analysis resources and antibiotic availability (i.e., a lack of microbiology tests was associated with a lower availability of antibiotics). Although some institutions cannot perform all microbiology analyses, they should have antibiotics available to avoid treatment delays since the time from infection to antibiotic use is associated with sepsis outcomes.^(24)^

We also evaluated ICU staffing in our sample, with encountered values lower than those observed in high-income countries^(25)^ and even Mongolian centers.^(9)^ Unfortunately, there is a paucity of current ICU staffing data from LMICs and its relationship with outcomes. Previous information has demonstrated the association between both nurse staffing^(26)^ and the intensivist-patient ratio^(27)^ with hospital mortality and severe burnout,^(28)^ but these data are mainly from high-income countries. In Brazil, Tironi et al. observed a burnout prevalence of 61.7% in intensivists and the lack of resources as a stressor during ICU shifts in 47.4% of staff.^(29)^ Recently, the ORCHESTRA study failed to demonstrate a significant impact of physician or nurse staffing patterns on hospital mortality in Brazil.^(30)^ Although we acknowledge that the ORCHESTRA study was not meant to specifically address septic patients and some differences between participating units in the ORCHESTRA and our study exist (such as the number of participating units from the private health system, geographic distribution alongside Brazilian regions and capitals, the nurse/patient ratio), we also did not identify staffing pattern as a significant factor associated with hospital mortality (Supplementary web appendix and Table 5 published with the SPREAD study - Lancet Infect Dis. 2017;17(11):1180-9).^(14)^

Our study has some strengths. Our sampling was representative of Brazilian institutions with ICUs. Our study design is original and might help explain the dynamics of resource availability in upper middle-income countries and may help plan future studies at the national level. The low rate of refusal to participate also improves our internal and external validity.

This study also has some limitations. First, the survey was self-reported, and we did not perform audits to evaluate whether the responses were accurate. However, the questionnaire was required to be fully completed before the units could participate in the SPREAD study, and the random stratified sampling method increases the validity and representativeness of our results. Second, although the questionnaire was designed by a committee with previous experience in critical care research and ICU organization aspects and reviewed by board-certified intensivists involved with ICU management, no assessment of test-retest reliability was performed. Third, our data might not be applicable to other countries, even LMICs, although the methods might be replicable in other countries to obtain high-quality data.^(4)^

## CONCLUSION

Our nationwide, randomized survey of a representative sample of Brazilian adult intensive care units indicates that in a substantial number of institutions, there is a lack of required resources to perform basic monitoring and interventions in septic patients. Our results highlight major opportunities for the improvement of effective evidence-based interventions in Brazil. This study may also serve as a framework to evaluate resource availability in low- and middle-income countries.
