# Supplementary material for: Availability of resources to treat sepsis in Brazil: a random sample of Brazilian institutions
Source: Rev Bras Ter Intensiva. 2019 Apr-Jun;31(2):193–201. doi: 10.5935/0103-507X.20190033 (PMC6649213; doi:10.5935/0103-507X.20190033)
Supplement: Supplementary file 1 [file rbti-31-02-0193-suppl1.pdf]

Availability of resources to treat sepsis in Brazil: a random sample of Brazilian institutions

Disponibilidade de recursos para tratamento da sepse no Brasil: uma amostra aleatória de instituições brasileiras

Leandro Utino Taniguchi[1,2,3], Luciano Cesar Pontes de Azevedo[1,2,3,4], Fernando Augusto Bozza[3,4,5,6], Alexandre Biasi Cavalcanti[3,4,7], Elaine Maria Ferreira[4], Fernanda Sousa Angotti Carrara[4], Juliana Lubarino Sousa[4], Reinaldo Salomão[4,8], Flávia Ribeiro Machado[3,4,9]; on behalf of the SPREAD Investigators and the Latin American Sepsis Institute Network

Intensive Care Unit (ICU) characteristics

*Required

General information*

Hospital name:

City and state where the unit is located:*

Most of ICU beds are dedicated to patients from (one option)

○ SUS
○ Private health system

Hospital characteristics (DATASUS):*

○ Public (Federal, State or Municipal)
○ Private philanthropic
○ Private non-philanthropic

Your hospital is (teaching status):*

○ University (has Medicine graduation course)
○ Non-university

Number of hospital beds:*

Number of beds in your ICU:*

Nurse/beds: How many nurses per patients do you have in your unit?*

Nurse/beds: How many nursing attendant per patients do you have in your unit?

Doctor/beds: How many doctors per patients do you have DURING THE DAY?*

Doctor/beds: How many doctors per patients do you have DURING THE NIGHT?*

HOSPITAL FACILITIES*

Does your hospital have?

	Yes	No	I don't know
Emergency	○	○	○
Operating theater	○	○	○
Own blood bank	○	○	○
Own laboratory	○	○	○
Own microbiology	○	○	○

DRUGS/MEDICATION*

Are the following medications available in your hospital?

	Always	Most of the times	In the minority of times	Never	I don't know
Ceftriaxone or ceftazidime	○	○	○	○	○
Cefepime	○	○	○	○	○
Piperacillin/tazobactan	○	○	○	○	○
Vancomycin or teicoplanin	○	○	○	○	○
Linezolide	○	○	○	○	○
Macrolides (azithromycin EV or claritrhromycin EV)	○	○	○	○	○
Echinocandins (caspofungin, anidulafungin or micafungin)	○	○	○	○	○
Tigeciclin	○	○	○	○	○
Hidrocortisone EV	○	○	○	○	○
Saline, lactated ringer or any other crystalloid	○	○	○	○	○
Colloid (starchs or gelatins)	○	○	○	○	○
Albumin	○	○	○	○	○
Oxygen	○	○	○	○	○
Unfractionated heparin	○	○	○	○	○
Ranitidine or another H2 blocker receptor	○	○	○	○	○
Omeprazole or another proton pump inhibitor	○	○	○	○	○
Noradrenaline	○	○	○	○	○
Dopamine	○	○	○	○	○
Dobutamine	○	○	○	○	○
Adrenaline	○	○	○	○	○
Vasopressin	○	○	○	○	○
Midazolan	○	○	○	○	○
Propofol	○	○	○	○	○
Dexmedetomidine	○	○	○	○	○
Neuromuscular blockers	○	○	○	○	○
Fentanyl	○	○	○	○	○
Red blood cell within 6 hours	○	○	○	○	○

PATIENT MONITORING DEVICES*

Are the following variables available in your hospital?

	Always	Most of the times	In the minority of times	Never	I don't know
Temperature	○	○	○	○	○
Oxygen oximetry	○	○	○	○	○
Automatic non-invasive blood pressure	○	○	○	○	○
Invasive arterial pressure	○	○	○	○	○
Central venous pressure (CVP)	○	○	○	○	○
Non-invasive cardiac output	○	○	○	○	○
Pulmonary artery catheter	○	○	○	○	○
Continuous central venous saturation (Presep®)	○	○	○	○	○

Do you manage?*

	Always	Most of the times	In the minority of times	Never	I don't know
Patients in septic shock with invasive arterial pressure?	○	○	○	○	○
Patients in septic shock with central venous pressure?	○	○	○	○	○
Patients with hyperlactatemia with central venous pressure?	○	○	○	○	○
Fluid responsiveness with dynamics parameters?	○	○	○	○	○

LABORATORY*

Are the following resources available in your hospital?

	Always	Most of the times	In the minority of times	Never	I don't know
Direct microscopy/Gram	○	○	○	○	○
Blood culture	○	○	○	○	○
Culture of respiratory secretions (qualitative)	○	○	○	○	○
Culture of respiratory secretions (quantitative)	○	○	○	○	○
Urine culture	○	○	○	○	○
Blood gas analysis in 3 hours	○	○	○	○	○
Lactate in 3 hours	○	○	○	○	○
Complete blood counts	○	○	○	○	○
Creatinine	○	○	○	○	○
Bilirrubin	○	○	○	○	○
Prothrombin time (INR)	○	○	○	○	○
C-reactive protein	○	○	○	○	○
Procalcitonin	○	○	○	○	○

Do you manage?*

	Always	Most of the times	In the minority of times	Never	I don't know
Central venous saturation in septic shock?	○	○	○	○	○
Central venous saturation in hyperlactatemia?	○	○	○	○	○
Lactate in severe sepsis suspicious?	○	○	○	○	○

EQUIPMENTS AND RESOURCES*

Are the following resources available in your hospital?

	Always	Most of the times	In the minority of times	Never	I don't know
Bedside X-ray	○	○	○	○	○
Bedside ultrasound	○	○	○	○	○
Bedside echocardiography	○	○	○	○	○
Computed tomography	○	○	○	○	○
Non-invasive ventilation	○	○	○	○	○
Invasive mechanical ventilation	○	○	○	○	○
Infusion pump	○	○	○	○	○
Peritoneal dialysis	○	○	○	○	○
Bedside hemodialysis	○	○	○	○	○

DISPOSABLES*

Are the following disposables available in your hospital?

	Always	Most of the times	In the minority of times	Never	I don't know
Urinary catheter	○	○	○	○	○
Peripheral catheter	○	○	○	○	○
Enteral tube feeding	○	○	○	○	○
Endotracheal tube	○	○	○	○	○
Oxygen mask	○	○	○	○	○
Oxygen nasal probes	○	○	○	○	○
Intracath catheters	○	○	○	○	○
Central venous catheter	○	○	○	○	○
Dialysis catheter	○	○	○	○	○

PROTOCOLIZED CARE*

Are the following protocols available in your hospital?

	Yes, a managed protocol	Yes, but not managed	No	I don't know
Sepsis	○	○	○	○
Glycemic control	○	○	○	○
Sedation/analgesia	○	○	○	○
Mechanical ventilation weaning	○	○	○	○
Nutrition support	○	○	○	○
